# Evaluating Psychological Interventions for Pain, Recovery, and Outcomes in Surgical and Medical Care

**DOI:** 10.7759/cureus.88097

**Published:** 2025-07-16

**Authors:** Ali Khalid, Ata Mohajer-Bastami, Ameer Khamise, Sjaak Pouwels, Sarah Moin, Vithusana Araamuthan, Aya Khalid, Ahmed Ahmed, Suhaib Ahmad

**Affiliations:** 1 School of Medicine, The University of Buckingham Medical School, Buckingham, GBR; 2 Department of Surgery, Ealing Hospital, London, GBR; 3 Stoke Mandeville Hospital, Buckinghamshire Health Trust, Aylesbury, GBR; 4 Intensive Care Medicine, Elisabeth-Tweesteden Hospital, Tilburg, NLD; 5 Department of General Surgery, East Surrey Hospital, London, GBR; 6 Department of Psychology, University of London, London, GBR; 7 School of Medicine, Medipol University International School of Medicine, Istanbul, TUR; 8 Department of General Surgery, Imperial College London, London, GBR; 9 Department of General Surgery, Ysbyty Gwynedd Hospital, Bangor, GBR

**Keywords:** anesthesia, hypnosis, non-pharmacological interventions, pain management, surgery

## Abstract

Hypnosis is a recognised technique in pain management and anaesthesia. Historically used by James Esdaile in the 19th century, it remains underutilised in modern clinical settings. This narrative review examines the historical context, mechanisms, and clinical applications of hypnosis, highlighting its effectiveness in general surgery, orthopaedics, urology, obstetrics, oncology, and paediatrics. Evidence shows that hypnosis can reduce perioperative pain, anxiety, and opioid use, while enhancing recovery. Neuroimaging studies reveal altered brain activity during hypnotic states, supporting its physiological basis. Despite these benefits, adoption remains limited due to misconceptions, lack of awareness, and insufficient training among healthcare providers. Cultural attitudes and the absence of standardised protocols also hinder its integration. Hypnosis has demonstrated particular value in chronic pain conditions such as fibromyalgia, irritable bowel syndrome, and cancer-related symptoms, offering a low-risk, cost-effective adjunct to care. This review calls for structured training to integrate hypnosis into routine, patient-centred clinical practice.

## Introduction and background

Hypnosis is a psychotherapeutic technique that can modulate awareness, relax the human body, and reduce anxiety [1,2]. It has been employed in medical contexts for over a century, initially popularised by James Esdaile, who conducted over 300 major surgeries using mesmerism alone as anaesthesia [3]. The practice of hypnosis has evolved considerably, from being dismissed as "fringe" medicine to gaining recognition as an effective, non-invasive intervention for pain relief and anxiety reduction in medical and surgical settings [4]. The accredited College of Medicine and Integrated Health in the UK recognises hypnosis and hypnotherapy as a helpful method of dealing with a wide range of conditions, including chronic pain, anxiety, and irritable bowel syndrome (IBS) [5].

Despite growing evidence of hypnosis in pain management, multiple barriers still remain for its implementation in routine clinical practice [6]. The biggest barriers identified seem to be regarding ongoing misconceptions about hypnosis and lack of confidence in implementing hypnosis successfully, as a result leading to reduced uptake of training and thus less delivery. Despite growing evidence, the lack of perceived "robust" empirical validation that is required for evidence-based research further limits its validation in modern clinical practice [7].

Recent systematic reviews and meta-analyses have validated hypnosis as an effective tool in pain management and anaesthesia [8,9]. These studies highlighted significant reductions in postoperative pain and decreased opioid consumption, supporting the incorporation of hypnosis into modern medical practice [8].

The aim of this narrative review is to provide a comprehensive overview of the historical and contemporary applications of hypnosis in pain management and anaesthesia in surgical settings. This paper will also consider more recent findings that explore the efficacy and mechanisms of action of hypnosis, as well as its integration into modern clinical practice. The incorporation of hypnosis into the treatment of medical conditions that benefit from its use will be discussed, along with consideration of how it can be used effectively to control symptoms beyond pain. The challenges and ethical considerations associated with hypnosis will also be addressed. Finally, possible future directions for its use as a more frequently applied adjunct in complex pain scenarios will be explored.

## Review

Methodology

A narrative review was conducted using Ovid MEDLINE, Scopus, and Google Scholar to identify relevant literature on the use of hypnosis in pain management, anaesthesia, and surgical or medical care. The following keywords and MeSH terms were used in combinations: “hypnosis,” “hypnotherapy,” “pain management,” “surgical procedures,” “perioperative care,” “chronic pain,” “psychological interventions,” and “anaesthesia.” Boolean operators (AND, OR) were applied to refine the results.

Inclusion criteria

Inclusion criteria included English-language articles; studies discussing clinical applications, mechanisms, or outcomes of hypnosis in medical or surgical settings; and case reports, randomised trials, meta-analyses, and narrative or systematic reviews.

Exclusion criteria

Exclusion criteria included non-English publications, articles without full text, and studies not focused on clinical use (e.g., recreational hypnosis).

History of hypnosis

Franz Anton Mesmer brought the hypnotic phenomenon into medical use among European practitioners in the 18th century [10]. He believed in a magnetic field surrounding and extending from individuals, which could be influenced to heal illness. James Braid, a surgeon and philosopher, observed a neurophysiological variant of sleep, whereby patients appeared asleep yet remained hyperalert. His work, alongside that of Alexandre Bertrand, laid the foundation for the modern explanation of the hypnosis phenomenon [10]. The term “hypnosis” derives from the Greek word hypnos, meaning sleep [11]. An earlier definition recognised hypnosis as “a subjective state in which alterations of perception or memory can be elicited by suggestion” [12], a definition that still holds relevance today.

James Esdaile was among the earliest practitioners of hypnosis for anaesthesia, reporting successful outcomes in over 300 surgical cases during the mid-19th century [3]. His use of mesmerism, a precursor to modern hypnosis, significantly reduced surgical shock and postoperative mortality rates. Despite his successes, the mainstream medical community largely ignored Esdaile’s work until the 20th century. In the mid-20th century, researchers such as Hilgard revitalised interest in hypnosis as a clinical tool, expanding its applications to obstetrics, dentistry, and chronic pain management [13]. Their work established a foundation for modern clinical hypnosis, demonstrating its effectiveness in reducing both pain and anxiety across various medical contexts. It was not until 1955 that anaesthetists, who had previously paid little attention to hypnosis due to the rapid adoption of inhaled anaesthesia, became more open to its use. That year, the British Medical Association declared that “there is a place for hypnotism in the production of anaesthesia or analgesia for surgery and dental operations,” a position supported by expert consensus [14], and followed three years later by the American Medical Association.

This paper recognises that different cultural perspectives can both aid in conceptualising how hypnosis functions and contribute to the broader debate surrounding the very definition of hypnosis [15]. There is true value in appreciating how cross-cultural differences have led to varying adoptions of hypnosis and hypnotic-like phenomena. Western approaches to hypnosis, often framed as etic, that is, universally applicable, may lead to varying levels of acceptance due to the nature of how they are represented. In contrast, non-Western cultures, both historically and in contemporary practice, often recognise hypnotic-like healing processes rooted in their own social constructs. One should consider these differing perspectives to develop a more universal understanding of hypnosis and how it can be broadly utilised [16].

Proposed mechanisms of hypnosis

Michael Heap, a former leading clinical and forensic psychologist, recognised hypnosis as a “complex psychological phenomenon” [17]. He later defined hypnosis as “a waking state of awareness, in which a person’s attention is detached from his or her immediate environment and is absorbed by inner experiences such as feelings, cognition and imagery.”

The state of hypnosis, therefore, can be thought of as a particular mental state in which an individual has reduced peripheral awareness but greater focused attention, colloquially referred to as a “trance.” This hypnotic trance, or altered mental state, can be either self-induced or induced by an external therapist. It is during this state that an individual may modify psychological and cognitive processes, as well as their behaviour [18].

Until recently, one argument against the objective effectiveness of hypnosis was the absence of measurable neurobiological markers during the hypnotic state. However, more recent evidence has guided researchers towards understanding novel concepts regarding the neural basis of hypnosis [19]. One comprehensive review found that, with the use of Magnetic Resonance Imaging (MRI) and electroencephalography (EEG), there is likely a causal relationship between functional connectivity and cognitive and behavioural responses [20]. Future research could explore how the functioning of the frontal lobes may be influenced by hypnotic states. Another recent study using EEG techniques in patients with fibromyalgia showed further promise [21]. The researchers demonstrated that neural oscillations during the hypnotic state were characterised by increased theta power in the left parietal and occipital electrodes, increased beta power in the frontal and left temporal electrodes, and increased slow-gamma power in the frontal and left parietal electrodes. These promising results suggest a possible electrophysiological marker of hypnosis, thus further strengthening the evidence base.

Other recent advances in neuroimaging have provided valuable insights into the mechanisms underlying hypnotic analgesia. Studies using functional MRI and positron emission tomography (PET) scans have shown that hypnosis alters activity in brain regions such as the anterior cingulate cortex, thalamus, and prefrontal cortex, all of which are involved in pain processing [22]. Faymonville et al. demonstrated that hypnosis can modulate both the sensory and affective components of pain, reducing the subjective experience without pharmacological intervention [4]. Pathak et al. used functional MRI to further explore how hypnosis modulates pain perception. Their study showed reduced activity in pain-processing areas of the brain, such as the somatosensory cortex, when patients were in a hypnotic state, thereby providing additional neurophysiological evidence for the effectiveness of hypnotic analgesia [23].

Application of hypnosis in surgery

Montgomery et al. conducted a meta-analysis of 20 well-controlled studies, concluding that hypnosis is an effective adjunctive treatment for surgical patients [24]. Patients who received hypnosis demonstrated reduced anxiety, decreased analgesic use, faster recovery times, and better overall outcomes compared to those who did not receive hypnosis. Chen et al. [25] studied the use of hypnosis in cataract surgery and found that hypnosis combined with topical anaesthesia improved patient cooperation and reduced pain and anxiety. However, it could not eliminate pain in all cases. The study suggested that individual differences in hypnotisability could impact the effectiveness of hypnotic interventions.

A recent meta-analysis conducted by Jones et al. further confirmed that pre-surgical hypnosis led to significantly lower levels of perioperative anxiety and reduced the need for opioid analgesics in patients undergoing surgery [8]. Additionally, McKernan et al. found that integrating hypnosis as part of the pre-surgical preparation reduced hospitalisation times and improved patient-reported outcomes, highlighting the broad utility of this approach [26].

There have been mixed outcomes in reviewing whether hypnosis is effective in monitored anaesthesia care. An older non-randomised study found no difference in outcomes between the study and control groups in patients being read a hypnosis script prior to undergoing radial keratotomy [27]. In contrast, more recent studies documented significant benefits from both pre-operative and intra-operative hypnosis [28,29], as well as in children undergoing painful invasive medical procedures [30]. Notably, these studies incorporated randomisation into their design. One could argue, therefore, that they have a "stronger" evidence-based backing and are more valid as a result. A further argument is that papers written longer ago, in a time when there was fear of an author's name becoming disreputable in their field if they veered away from the norm of clinical practice, may have led to a degree of subconscious researcher bias. 

Application of hypnosis in surgical specialities

Orthopaedic Surgery

Fathi et al. reported on a case study involving hypnosis as an alternative to general anaesthesia for orthopaedic hand surgery [31]. The patient, a 32-year-old male, underwent successful hand surgery using hypnosis without the need for pharmacological intervention. The patient’s vital signs remained stable throughout the procedure, and postoperative pain was minimised without the use of analgesic drugs, indicating the potential of hypnosis as a reliable anaesthetic alternative. The previously mentioned study reviewing patients undergoing hand surgery also found decreased pain in those who underwent hypnosis, as well as fewer postoperative complications [1].

Another wider study showed similar results. A recent randomised controlled trial investigating the use of peri-operative hypnosis in patients undergoing knee arthroplasty discovered a significant reduction in inpatient opioid use in patients who had undergone hypnosis [32].

Urology

A case review of a patient who developed secondary diurnal enuresis following a traumatic road traffic accident explored the outcome of using hypnosis as a treatment. The result showed that the patient was symptom-free after six months of follow-up [33]. Another case review evaluated the effectiveness of hypnosis in alleviating pain associated with vulvar vestibulitis syndrome. The patient remained pain-free following treatment and continued to do so at a 12-month follow-up [34]. Further to this, Freeman and Baxby conducted a study on women with proven detrusor instability leading to incontinence. They found that, particularly for ‘idiopathic’ detrusor instability, psychological factors played a significant role and hypnosis proved to be an effective tool for the disorder [35].

Obstetric Surgery

Ralph August utilised medical hypnosis in 1957-1958 to facilitate the delivery of 295 babies, with 94% of the cases relying solely on hypnosis as anaesthesia [36]. The remaining cases required additional local or general anaesthesia. August emphasised the reduction in anaesthesia-related risks for both mother and child and highlighted the improved relationship between patient and practitioner. More recent studies, such as those by Werner et al., conducted randomised controlled trials on 1,222 first-time mothers, showing that self-hypnosis led to better childbirth experiences and reduced the need for emergency interventions [37,38]. Brown and Hammond [39] investigated the effectiveness of hypnosis during labour and demonstrated a significant reduction in the use of anaesthesia and analgesic agents, as well as shorter stages 1 and 2 of labour. Other benefits, including prolonging pregnancy and thereby reducing the number of preterm births, along with decreased use of analgesics, were shown in a further case-control study [40].

General/Abdominal Surgery

Villa et al. conducted a systematic review on the effectiveness of psychological interventions in the peri-operative period, including hypnosis as well as cognitive-behavioural therapies and relaxation techniques. They found all interventions to be effective in improving patients’ anxiety and pain outcomes peri-operatively [41].

Galili et al. aimed to determine whether hypnosis could be an effective method for children with functional chronic recurrent abdominal pain of unknown aetiology [42]. Not only could this reduce the burden of unnecessary laparoscopic surgery, but among 17 patients who underwent a single session of hypnosis, 14 reported full symptom resolution. This is extremely promising, as it widens the potential use of hypnosis beyond adult populations, particularly since the researchers concluded that no residual side effects were reported during the follow-up period in these children.

A retrospective case-control study evaluating the use of hypnosis alongside local anaesthesia during endovascular abdominal aneurysm repair (EVAR) as an alternative to general anaesthesia found that hypnosis is not only feasible and safe, but also resulted in lower intraoperative norepinephrine use, shorter procedure duration, and reduced postoperative hospital stay [43]. This shows promise not only in enhancing patient safety but also as an argument for cost-effectiveness in this and other surgical contexts.

Role of hypnosis in medical specialities and pain management

The National Institutes of Health Consensus Development Programme further affirmed the strength of evidence supporting hypnosis for chronic pain [44]. Even today, the previously mentioned College of Medicine recognises the positive impact that hypnosis can have on a range of chronic conditions [5].

Issues related to oncology patients could certainly pertain to pain. Side effects of treatments such as chemotherapy and radiotherapy can be a huge burden on patients, which in turn can lead to further stress and anxiety. A meta-analysis showed the effectiveness of hypnosis in alleviating anxiety related to cancer in patients [45]. As briefly mentioned previously, hypnosis is not limited to adults. Richardson et al. conducted systematic reviews on how effective hypnosis can be for procedure-related pain in children with cancer, as well as alleviating nausea and vomiting symptoms induced by chemotherapy [46,47]. They concluded in both cases that hypnosis can reduce the respective symptoms; however, they did acknowledge some methodological limitations and recommended future research in the field. Fatigue related to radiotherapy has also been found to be improved by the use of hypnosis [48].

IBS has been a recognised condition that has improved from hypnotherapy in a number of studies [49,50], and an argument can be made on embedding hypnosis into the routine management for IBS, both due to its shown efficacy as well as from a cost-effectiveness perspective compared to currently available treatment.

Critical reviews of the use of hypnosis in asthma [51,52] had previously shown how hypnosis can be an effective treatment for reducing the severity of symptoms related to asthma and can be particularly useful in improving the emotional state that can worsen airway obstruction; this was particularly helpful for children. The studies were limited in both their sample size and the degree of susceptibility of patients to reach a hypnotic state.

All the above demonstrate that hypnosis goes well beyond just managing acute or chronic pain. That said, generally more research has been conducted on the effects hypnosis can have on chronic pain; other chronic conditions and their respective symptoms should still be considered going forward. Complex pain-related conditions such as fibromyalgia tend to have a significant economic burden on healthcare systems globally [53], as well as a wider societal impact. Aravena et al. [54] conducted a randomised controlled trial. The results showed the positive impact hypnosis can have in significantly decreasing the intensity of pain and fatigue in patients with fibromyalgia, with the added benefit of reduced depressive symptoms. Other cases of complex pain, such as migraines, have been shown to be improved with hypnosis as well as acupuncture, so much so that it can be compared as equivalent to current treatment [55]. One can wonder why hypnosis is not considered, at the very least, as an adjunct to complex chronic pain in multidisciplinary team discussions, given its cost-effectiveness and lack of risks.

Implementation challenges of hypnosis

Despite the promising evidence supporting the use of hypnosis in clinical practice, several challenges remain. Individual hypnotisability variability can affect the efficacy of hypnosis, making it difficult to standardise protocols. Additionally, there is a lack of formalised training for medical professionals, which limits the widespread adoption of hypnosis in healthcare settings [56]. Together with better training, having a more standardised regulation or regulatory body, as is the case for clinicians such as the General Medical Council for UK doctors, would likely ensure that the medical profession, as well as the general public, considers this a more acceptable form of practice.

As previously stated, hypnosis is limited by the susceptibility of the individual to enter a hypnotic state, which was a limitation in some of the aforementioned studies. Generally, patients who are more receptive to hypnosis will gain more benefit in pain reduction compared to those who are less receptive [10].

Another barrier to implementing hypnosis as a treatment is the lack of perceived robust research. It is unfortunate that a wide-scale study would require a significant time investment and commitment from the research team and participants, which limits its appeal for funding compared to, for example, a trial for a novel cancer medication. That said, medical commissioners are unlikely to be convinced to invest in this field without a stronger evidence base. Despite existing and growing research, more methodologically robust studies are needed [7].

Discussion for future directions

Future research should focus on developing standardised protocols tailored to individual patient needs to maximise the benefits of hypnosis in clinical practice. Training healthcare professionals in hypnotic techniques will ensure consistency and effectiveness. Furthermore, larger-scale clinical trials are required to validate the findings from case studies and smaller trials, paving the way for the broader adoption of hypnosis as a complementary tool in pain management.

Recent studies underscore the importance of individualised hypnosis protocols tailored to specific patient characteristics, such as hypnotisability and type of surgery. Future research should focus on optimising these protocols and investigating their effectiveness across diverse patient populations and surgical procedures. Moreover, training programmes for healthcare professionals on the application of hypnosis in clinical settings should be standardised to ensure consistency and quality of care [26].

## Conclusions

Hypnosis has recently spiked the interest of medical practitioners around the world. In different cultures, it has been accepted either as a sole treatment with a more Ayurvedic approach or as an adjunct for multiple conditions. There does exist an evidence base for its effectiveness, which goes beyond being anecdotal. However, as with many novel treatments, implementation proves to be challenging. Since hypnosis is risk-free and has shown effectiveness for a range of conditions and for pain management, this paper would argue that more consistent and frequent use of hypnosis should be considered by clinicians.
